# Peripherally inserted central catheters versus implanted port catheters in patients with breast cancer: a *post hoc* analysis of the PICCPORT randomised controlled trial

**DOI:** 10.1016/j.bjao.2025.100377

**Published:** 2025-02-04

**Authors:** Anton Utas, Stefanie Seifert, Knut Taxbro

**Affiliations:** 1 Department of Anesthesia and Intensive Care Medicine, Ryhov County Hospital, Jönköping, Sweden; 2 Department of Biomedical and Clinical Sciences, Linköping University, Linköping, Sweden

**Keywords:** breast neoplasms, catheterisation, catheter-related infections, central venous, central venous catheters, patient satisfaction, upper extremity deep vein thrombosis, vascular access devices

## Abstract

**Background:**

Breast cancer is the most prevalent malignancy affecting women. However, the optimal strategy for patients requiring long-term central venous catheters in breast cancer treatment remains uncertain. Previous investigations involving a mixed cancer population have shown a higher frequency of adverse events among patients receiving peripherally implanted central catheters (PICCs) compared with totally implanted central catheters (PORTs). Our study aimed to compare catheter-related adverse events in breast cancer patients.

**Methods:**

We conducted a *post hoc* analysis of a previously published multicentre RCT known as PICCPORT. Data pertaining to baseline characteristics, insertion specifics, complication rates, and patient satisfaction were collected for breast cancer patients who required long-term central venous catheters for cancer treatment. The primary endpoint was a composite variable encompassing thrombotic, occlusive, infectious, or mechanical complications, while patient satisfaction served as a secondary endpoint.

**Results:**

Our analysis included 80 patients receiving PORT and 78 patients receiving PICC. There was no statistically significant difference in the incidence of complications between the PICC and PORT groups. Interestingly, PICC insertion was less painful than PORT insertion, although both groups reported low levels of pain.

**Conclusions:**

While acknowledging the limitations of an underpowered *post hoc* subgroup analysis, our findings suggest that the well-established superiority of PORTs in terms of adverse events among cancer patients might not be as substantial for breast cancer patients in particular. Ultimately, the optimal strategy for selecting long-term access devices in breast cancer patients remains to be determined.

**Clinical trial registration:**

NCT01971021.

Breast cancer is the most prevalent malignant disease affecting women worldwide, with the highest incidence in women between 60 and 69 yr of age.^1^^,^^2^ Notably, survival rates for breast cancer are higher than those for many other malignancies.^2^ This favourable outcome may be attributed to factors such as early diagnosis, a broader array of treatment options, and lower comorbidity rates.^3^

Systemic chemotherapy for breast cancer often necessitates the use of long-term central venous catheters (CVCs). Two common types of long-term CVCs are peripherally inserted CVCs (PICCs) and fully implanted CVCs (PORTs). In Sweden, PORTs are typically implanted by anaesthetists in an operating theatre, whereas PICCs are more commonly inserted at the patient's bedside by oncology nurses.^4^^,^^5^ The increasing adoption of PICCs is driven by perceived safety, limited theatre capacity, and the goal of reducing costs and waiting times.^6^

A number of complications are associated with long-term CVCs, including pneumothorax, bleeding, thrombosis, catheter-related infections, and catheter occlusion. These complications not only contribute to increased morbidity and mortality, but can also lead to delays in cancer treatment.^7^^,^^8^ PICCs appear to carry a higher risk of developing catheter-related thrombosis compared with PORTs, particularly among cancer patients.^8^, ^9^, ^10^, ^11^, ^12^, ^13^ Conversely, catheter-related infections occur at similar rates for both PICCs and PORTs.^9^^,^^14^^,^^15^ RCTs involving patients with various malignancies consistently demonstrate fewer complications overall among patients receiving PORTs compared with PICCs.^10^^,^^16^^,^^17^ Additionally, there are indications that healthcare costs could be lower and patient satisfaction higher among patients receiving PORTs compared with PICCs.^16^^,^^17^

Among breast cancer patients, retrospective studies and one RCT have shown a higher incidence of complications among those receiving PICCs compared with PORTs.^11^^,^^18^ However, it is worth noting that the total number of complications appears to be lower in breast cancer patients compared with the broader group of all cancer patients.^11^ The mounting evidence suggests that PORTs may indeed offer superiority over PICCs. Furthermore, findings from studies involving diverse malignancies may not be directly applicable to breast cancer patients.

Given that breast cancer is the most prevalent cancer among women, it becomes imperative to delineate optimal vascular access strategies specifically tailored to this population.^1^^,^^2^ Consequently, our study aims to conduct a subgroup analysis of breast cancer patients included in the PICCPORT trial, aiming to provide valuable insights into this critical aspect of clinical practice.^17^

## Methods

### Patient population and inclusion/exclusion criteria

Patients aged ≥18 yr with a diagnosis of non-haematological cancer, a life expectancy exceeding 4 weeks, and a requirement for chemotherapy via a long-term CVC were considered eligible for inclusion in the original PICCPORT trial. However, patients meeting any of the following criteria were excluded: if there was an ongoing severe systemic infection, a clinically significant upper extremity or central deep vein thrombosis (DVT), severe coagulopathy, inability to communicate, or an imminent need for a dialysis fistula.

The patient population for the *post hoc* subgroup analysis was extracted from the PICCPORT trial and comprised all patients with breast cancer.

### Ethical considerations

The PICCPORT trial received approval from the Regional Ethics Committee in Linköping (reference number: EPN 2013/56-31) and adhered to the principles outlined by the International Council for Harmonisation – Good Clinical Practice and the Helsinki Declaration. The study also followed the Consolidated Standards of Reporting Trials (CONSORT) methodology.

During data analysis, patient identification was coded to ensure confidentiality. Data sheets were securely stored on a server provided by Region Jönköping County. Access to the server was restricted through password protection, allowing only the research team to retrieve and analyse the data. Importantly, all study participants provided written consent before their inclusion in the trial.

### Conduct of the study, measurements, and data handling

Eligibility screening for patients subsequently included in the PICCPORT trial was conducted by nursing staff at both inpatient and outpatient clinics. Those meeting the inclusion criteria were subsequently informed and enrolled by a physician from the Department of Oncology. The randomisation sequence was generated by a computer and organised by an independent statistician, using a block size of four and considering stratification to the two recruiting centres. Because of the unique characteristics of the catheters involved, blinding of patients, clinicians, or trial assessors to their assigned treatment arm was not feasible. Data were collected and recorded by the clinical trial unit staff at various time points: randomisation, post-catheter implantation, and during follow-up assessments at 1, 3, 6, and 12 months. For conduction of this *post hoc* analysis, no further data were collected. Available data sheets from the PICCPORT trial were used to extract data regarding patients with breast cancer.

### Patient satisfaction assessment

Patient satisfaction was evaluated in the PICCPORT trial through a questionnaire administered on the day of access device insertion, and also at 1, 6, and 12 months thereafter. The questionnaire aimed to assess the level of discomfort experienced during access device insertion and its subsequent impact on daily activities. A numerical rating scale (NRS) was utilised to determine pain intensity, with patients grading their pain on a scale from 0 (no pain) to 10 (worst pain imaginable).

### Definitions

#### Catheter-associated infections

These were defined in accordance with criteria established by the Infectious Diseases Society of America.^19^ Pocket, tunnel, or exit-site infection was defined as local signs of inflammation with culture-positive fluid obtained from the pocket, tunnel, or catheter exit-site. Catheter-related blood stream infection (CRBSI) was defined as clinical signs of systemic infection together with the isolation of the same microorganism from the tip culture and a blood culture drawn from another vessel, or through differential time to positivity cultures. All these types of infections were classified as a type of catheter-associated infection and the type of catheter-associated infection was not specified in the data analyses.

#### Catheter-related deep vein thrombosis

Criteria (i): presence of symptoms or clinical signs indicative of a DVT (e.g. pain, redness, swelling, tenderness) in a relevant area, confirmed through ultrasound or CT imaging. Criteria (ii): incidental detection of a DVT during imaging conducted for other medical reasons.

The decision to proceed with imaging based on clinical signs was at the discretion of the treating physician. Regular imaging for silent thrombosis was not part of the protocol.

#### Catheter occlusion

Catheter occlusion is characterised by the catheter's inability to be aspirated or flushed. It was considered in the analysis only if intervention with alteplase or ethanol instillation was necessary to resolve the occlusion.

#### Endpoints

The primary endpoint of this *post hoc* analysis was a clinically significant complication, defined as a composite endpoint encompassing catheter-related adverse events necessitating intervention. These events included thrombotic, occlusive, infectious, and mechanical complications. The secondary endpoint focused on patient satisfaction.

### Sample size calculation and statistical analyses

As this analysis was conducted *post hoc*, no formal sample size calculation was performed *a priori*. A *post hoc* power analysis regarding the primary endpoint was conducted. Various statistical tests were used for group comparisons, depending on the nature and distribution of the data. These tests included Pearson's χ^2^ test, Fisher's exact test, Mann–Whitney *U* test, and Student's *t*-test, as appropriate.

Analyses for primary and secondary outcomes in the breast cancer patient group were conducted on both the intention-to-treat and per-protocol populations. To identify independent predictors of the primary outcome and to visualise cumulative complication-free catheter survival (catheter-related adverse events during follow-up), including thrombosis, occlusion, infection, mechanical issues, or death, multivariable Cox proportional hazard models were utilised. Predictors with *P*-values greater than 0.05 were stepwise removed from the model.

All *P*-values were two-tailed, and a significance level of *P*<0.05 was considered statistically significant. Statistical analyses were conducted using SPSS version 27 (IBM, Armonk NY, USA).

## Results

**Fig 1:**
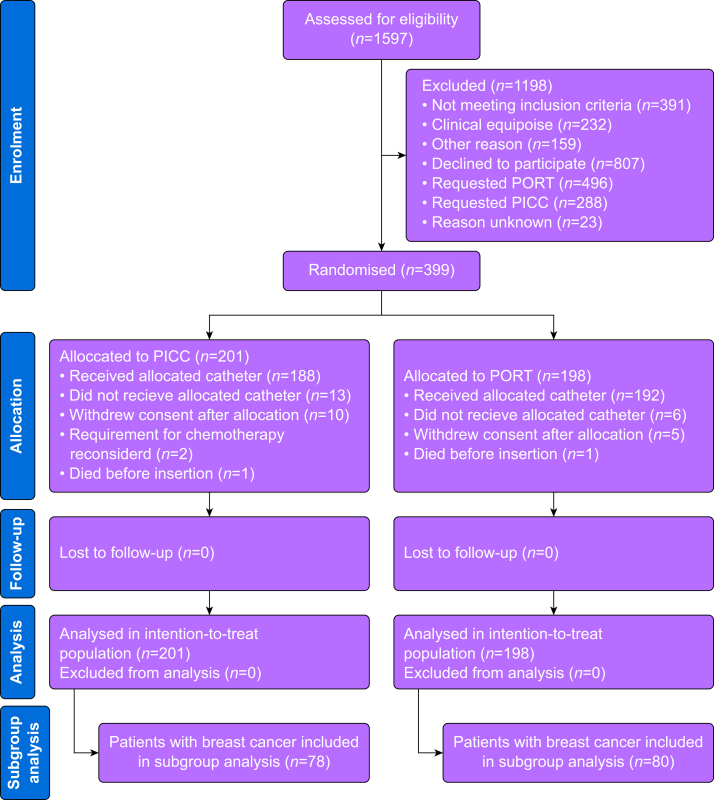
Study outline. No allocated patients were excluded during follow-up and analysis. PICC, peripherally inserted central catheter; PORT, totally implanted access port.

Study participants were recruited from two centres between 13 March 2013 and 16 February 2017. Data collection continued until 1 yr after the last patient was enrolled. A total of 1597 patients underwent eligibility assessment, out of which 399 patients were randomly allocated to either the PICC (*n*=201) or PORT (*n*=198) group. Among the allocated patients, 158 had breast cancer (40%), with 80 in the PORT group and 78 in the PICC group (Fig 1).

Baseline characteristics were similar in the two groups (Table 1). Notably, more antibiotics were administered in the PORT group than in the PICC group, and the procedure time during insertion was significantly shorter for the PICC group (Table 2).

**Table 1:** Baseline characteristics in the intention-to-treat population. Data are presented as *n* (%) or median (inter-quartile range). PICC, peripherally inserted central catheters; PORT, totally implanted access port.

	PICC (*n*=78)	PORT (*n*=80)
Sex		
Female	78 (100)	79 (99)
Male	0 (0)	1 (1)
Age	61 (53–69)	62 (53–68)
Cancer		
Breast	78 (100)	80 (100)
Treatment goal		
Adjuvant	73 (94)	78 (98)
Palliative	5 (6)	2 (3)

**Table 2:** Insertion characteristics and procedure-related complications in the per-protocol population. Data are presented as *n* (%) or median (inter-quartile range). LA, local anaesthetic; PICC, peripherally inserted central catheters; PORT, totally implanted access port; RA, right atrium; SVC, superior vena cava.

	PICC (*n*=74)	PORT (*n*=77)	*P*-value
Waiting time before insertion (days)	8 (6–12)	9 (7–14)	0.74
Inserting clinician			
Anaesthetist (in theatre)	0 (0)	67 (87)	
Surgeon (in theatre)	0 (0)	10 (13)	
Nurse specialist (in ward procedure room)	74 (100)	0 (0)	
Insertion vein			
Internal jugular	0 (0)	62 (81)	
Subclavian	0 (0)	3 (4)	
Brachial	5 (7)	0 (0)	
Basilar	67 (91)	0 (0)	
Cephalic	1 (1)	10 (13)	
Missing value	1 (1)	2 (3)	
Laterality			
Left	35 (47)	27 (35)	
Right	35 (47)	49 (64)	
Missing value	4 (5)	1 (1)	
Ultrasound guidance			<0.001
Yes	72 (97)	64 (83)	
No	1 (1)	12 (16)	
Missing value	1 (1)	1 (1)	
Fluoroscopy guidance			<0.001
Yes	0 (0)	71 (92)	
Missing value	0 (0)	6 (8)	
Puncture attempts	1 (1–1)	1 (1–2)	0.47
Arterial puncture	0 (0)	0 (0)	
Pneumothorax			
Suspected or verified	0 (0)	0 (0)	
Haematoma			
Intervention required	0 (0)	0 (0)	
Assistance from colleague required	0 (0)	1 (1)	1.00
Procedure failed	1 (1.4)	0 (0)	0.49
Antibiotics during the procedure	1	10	0.023
Anaesthetic approach			
No LA	1 (1)	0 (0)	
LA only	70 (95)	64 (83)	
LA and sedation	0 (0)	9 (12)	
General anaesthesia	0 (0)	1 (1)	
Missing value	3 (4)	3 (4)	
Tip position			1.00
Distal SVC/RA	69 (93)	71 (92)	
Other	5 (7)	6 (8)	
Procedure time (min)	20 (16–25)	29 (21–39)	<0.001

Regarding the primary endpoint, which encompassed all adverse events, there were 20 events in the PICC group and 14 events in the PORT group (Table 3). Furthermore, the PORT group had a higher number of catheter days per patient, resulting in a greater incidence of adverse events per 1000 catheter days in the PICC group. However, when adjusting for the number of catheter days, this difference was not statistically significant (*P*=0.114) based on the log-rank test.

**Table 3:** Dwell-time characteristics and complications in the intention-to-treat population. Data are presented as *n* (%) or median (inter-quartile range). *P*-values calculated using log-rank test, χ^2^ test, Mann–Whitney *U* test, and Fischer's exact tests, where appropriate. CD, catheter days; CR-DVT, catheter-related deep vein thrombosis; PICC, peripherally inserted central catheters; PORT, totally implanted access port.

	PICC (*n*=78)	PORT (*n*=80)	*P*-value
Catheter days per patient	127 (106–143)	211 (151–290)	<0.001
Total number of CD	9509	17 183	
All adverse events			0.11
All grade adverse events	20 (26)	14 (18)	
All grade adverse events per 1000 CD	2.10	0.81	
Days to all grade event	113 (90–141)	210 (148–290)	<0.001
Device removal			0.31
Patient died with device *in situ*	1 (1)	2 (3)	
Device removal as a result of adverse event	4 (5)	9 (11)	
End of treatment or follow-up	73 (94)	69 (86)	
DVT			
CR-DVT	5 (6)	2 (3)	0.23
CR-DVT per 1000 CD	0.53	0.11	
Device infection			
Device infection	2 (3)	9 (11)	0.033
Device infection per 1000 CD	0.21	0.52	
Catheter occlusion			
Catheter occlusion	11 (14)	1 (1)	0.002
Catheter occlusion per 1000 CD	1.16	0.059	
Mechanical event			
Mechanical event	2 (3)	2 (3)	0.98
Mechanical event per 1000 CD	0.21	0.11	

**Fig 2:**
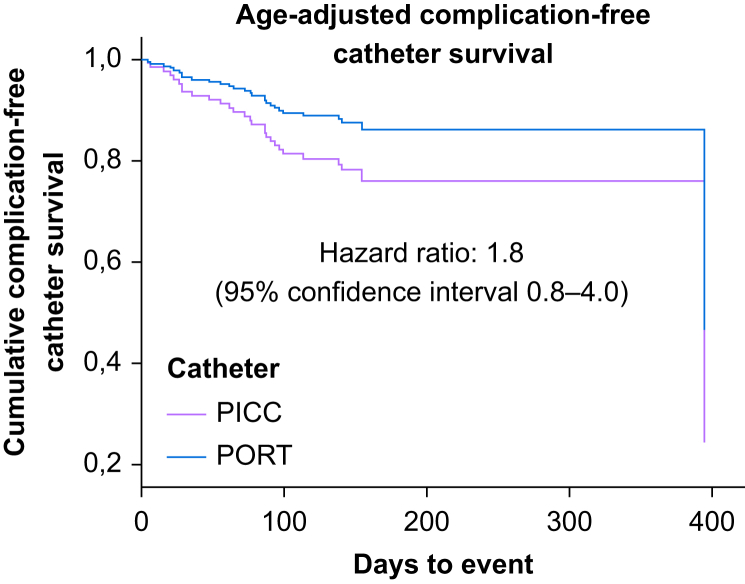
Age-adjusted complication-free catheter survival curve in the per-protocol (PP) population. PICC, peripherally inserted central catheter; PORT, totally implanted access port.

Figure 2 illustrates the cumulative age-adjusted adverse event-free catheter survival rates for both study groups. Among the adverse events in the PICC group, catheter occlusions were predominant (11/20). Notably, catheter occlusion occurred more frequently in the PICC group compared with the PORT group (*P*=0.002). Conversely, rates of device infection were higher in the PORT group than in the PICC group (*P*=0.033), with 9/14 adverse events in the PORT group being catheter-associated infections (predominantly local infections). No significant differences were observed for catheter-related DVT (CR-DVT) or mechanical events. Notably, five and two CR-DVTs occurred in the PICC and PORT groups, respectively. Analyses conducted in both the intention-to-treat and per-protocol populations yielded consistent results.

A *post hoc* power analysis was conducted regarding the primary endpoint. With an event rate of 20 and 14, respectively, there was a 17.6% chance of detecting a true difference between the two groups.

The proportion of patients reporting the implantation as painful in the PORT group was almost twice that of those in the PICC group (*P*<0.001). Although the median NRS pain score was low in both groups, a larger proportion of patients in the PORT group experienced an NRS pain score ≥4 during insertion (*P*=0.002). When questioned 3 months after insertion, significantly more patients in the PORT group reported an NRS pain score ≥4 during dressing change (for PICC) or needle insertion (for PORT) (*P*<0.001). Additionally, PORT caused more discomfort than PICC 1 month after insertion (*P*=0.022), although there was no difference after 3 months. Interestingly, the PICC group experienced more problems with the device when taking a shower (*P*<0.001) (see Supplementary Table S1). Patient satisfaction questionnaires at 6 and 12 months had very few respondents, so no further analyses were conducted.

## Discussion

The main finding of this trial suggests that there might not be a significant difference in adverse events between patients with breast cancer receiving PICC and those receiving PORT. Although there were fewer adverse events in the PORT group, the difference was not statistically significant. Notably, patients with PICC experience more problems during showering, whereas patients with PORT report more pain during device insertion. However, it is important to note that the level of pain associated with insertion, dressing changes, and needle insertions is low in both groups and may be clinically insignificant.

Our study contributes to the limited body of evidence from RCTs comparing adverse events in breast cancer patients receiving PICC *vs* PORT. Interestingly, our results somewhat contradict those of Clatot and colleagues,^11^ who found that adverse events were more common in patients receiving PICC. However, differences in the definition of adverse events between studies make direct comparison challenging. Unlike our study, Clatot and colleagues implemented a structured approach to device removal, which could have influenced the results by reducing passive catheter days. Additionally, adverse events occurred later in the PORT group compared with the PICC group in our study, raising questions about whether this is related to device characteristics or longer dwell time, which may be passive.

In a mixed cancer population, there is compelling evidence that adverse events occur more frequently in patients receiving PICC than in those receiving PORT.^10^^,^^16^^,^^17^ Although promoting PORT insertion may be appropriate for most cancer patients, it is essential to recognise that breast cancer patients differ from the general cancer population. Breast cancer boasts high survival rates, and the age of diagnosis tends to be relatively young.^20^ Additionally, diagnostic and therapeutic options for breast cancer diverge from those for other cancer types. Notably, breast cancer does not cause mucosal barrier injury, unlike gastrointestinal tract cancer, which may impact the risk of adverse events associated with long-term CVCs. The presence of fewer risk factors for adverse events in breast cancer patients could partially explain our study results. Furthermore, dwell-time data from the PICCPORT trial indicate that breast cancer patients had fewer catheter days than the non-breast cancer group (median dwell time 146 days [109–224] *vs* 154 [66–260], *P*=0.2).^17^ Therefore, the time at risk for catheter-related complications could be lower among patients with breast cancer compared with other malignancies.

This study has several limitations that warrant acknowledgement. Firstly, it constitutes an underpowered *post hoc* subgroup analysis of a larger trial. The subgroup was selected after randomisation, potentially introducing bias. Therefore, statistical results should be interpreted with this in mind. Secondly, the longer dwell time observed in the PORT group suggests that patients with PORTs may retain their devices for an extended period after treatment completion, potentially leading to a higher number of passive adverse event-free days in the PORT group. Thirdly, the study did not capture information on vein–catheter ratios, anticoagulation use, or other drugs during the follow-up period. Finally, routine radiological examinations were not conducted during the dwell time, leaving tip localisation, the presence of asymptomatic, undetected CR-DVT, and fibroelastic sleeves, uncertain.

In summary, this study reveals no significant difference in overall adverse events between patients receiving PICCs and those using PORTs for breast cancer treatment. Owing to the study's underpowered and *post hoc* nature, the clinical significance of the results is uncertain. However, given the substantial population of breast cancer patients, tailored recommendations could significantly influence upcoming CVC guidelines, complication rates, and patient satisfaction. Therefore, large-scale studies in specific cancer patient groups are essential to determine the optimal choice for long-term CVCs.

## Authors’ contributions

Study conception and design: all authors

Data acquisition: AU

Data analysis: AU, KT

Data interpretation: all authors

Manuscript drafting: AU

Critical revision of manuscript: SS, KT

Accountability of content: all authors

## Funding

Departmental funds (to AU); Futurum – The Academy for Health and Welfare, Region Jönköping County, Sweden (to KT and SS). The sponsor had no role in in study design, collection, analysis, and interpretation of data, writing the report, and the decision to submit the report for publication.

## Declaration of Generative AI and AI-assisted technologies in the writing process

During the preparation of this work, the authors used Microsoft® Copilot in order to improve language and readability. After using this tool, the authors reviewed and edited the content as needed and take full responsibility for the content of the publication.

## Declaration of interests

The authors declare that they have no conflicts of interest.
