# Supplementary material for: Peripherally inserted central catheters versus implanted port catheters in patients with breast cancer: a post hoc analysis of the PICCPORT randomised controlled trial
Source: BJA Open. 2025 Feb 4;13:100377. doi: 10.1016/j.bjao.2025.100377 (PMC11847134; doi:10.1016/j.bjao.2025.100377)
Supplement: Multimedia component 1 [file mmc1.docx]

Supplement 1: Patient satisfaction in the per-protocol population. Data presented as n (%) or median (interquartile range). P-values calculated using log rank test, χ²-test, Mann-Whitney U, and Fischer’s exact tests, where appropriate. PICC, Peripherally inserted central catheters. PORT, Totally implanted access port. NRS, numerical rating scale.

|  | **PICC (n = 74 )** |  | **PORT (n = 77)** |  | **P-value** |
| --- | --- | --- | --- | --- | --- |
|  |  | *Missing data* |  | *Missing data* |  |
| **Month 1 after insertion** |  |  |  |  |  |
| **I was worried before implantation** | 33 (45) | *2 (3)* | 39 (50) | *0 (0)* | 0,42 |
| **The implantation was painful** | 12 (16) | *2 (3)* | 28 (36) | *1 (1)* | < 0,001 |
| **NRS pain level ≥ 4 during insertion** | 9 (12) | *2 (3)* | 26 (34) | *0 (0)* | 0,002 |
| **NRS pain level ≥ 4 during dressing change (PICC)/Needle insertion (PORT)** | 5 (7) | *2 (3)* | 11 (14) | *0 (0)* | 0,15 |
| **Device causes discomfort** | 16 (22) | *2 (3)* | 26 (34) | *1 (1)* | 0,022 |
| **Device interferes when I take a shower** | 17 (23) | *2 (3)* | 5 (6) | *0 (0)* | < 0,001 |
| **…..I take a bath** | 4 (5) | *48 (65)* | 1 (1) | *39 (51)* | 0,10 |
| **…..I work out** | 3 (4) | *10 (14)* | 4 (5) | *7 (9)* | 0,24 |
| **…..I move my arm** | 9 (12) | *5 (7)* | 6 (8) | *1 (1)* | 0,15 |
| **…..I get dressed** | 6 (8) | *5 (7)* | 6 (8) | *1 (1)* | 0,63 |
|  |  |  |  |  |  |
| **Month 3 after insertion** |  |  |  |  |  |
| **NRS pain level ≥ 4 during dressing change (PICC)/Needle insertion (PORT)** | 4 (5) | *8 (11)* | 21 (27) | *3 (4)* | < 0,001 |
| **Device causes discomfort** | 10 (14) | *11 (15)* | 13 (17) | *5 (7)* | 0,47 |
| **Device interferes when I take a shower** | 8 (11) | *9 (12)* | 3 (4) | *3 (4)* | 0,002 |
| **…..I take a bath** | 2 (3) | *46 (62)* | 1 (1) | *34 (44)* | 0,11 |
| **…..I work out** | 1 (1) | *12 (16)* | 3 (4) | *9 (12)* | 0,70 |
| **…..I move my arm** | 4 (5) | *9 (12)* | 6 (8) | *3 (4)* | 0,84 |
| **…..I get dressed** | 5 (7) | *8 (11)* | 4 (5) | *3 (4)* | 0,059 |
